# The Influence of Ionic and Nonionic Surfactants on the Colloidal Stability and Removal of CuO Nanoparticles from Water by Chemical Coagulation

**DOI:** 10.3390/ijerph16071260

**Published:** 2019-04-09

**Authors:** Rizwan Khan, Muhammad Ali Inam, Sarfaraz Khan, Andrea Navarro Jiménez, Du Ri Park, Ick Tae Yeom

**Affiliations:** 1 Graduate School of Water Resources, Sungkyunkwan University (SKKU) 2066, Suwon 16419, Korea; rizwankhan@skku.edu (R.K.); aliinam@skku.edu (M.A.I.); enfl8709@skku.edu (D.R.P.); 2 Key Laboratory of the Three Gorges Reservoir Region Eco-Environment, State Ministry of Education, Chongqing University, Chongqing 400045, China; Sfk.jadoon@yahoo.com (S.K.); andrenavarrojimenez@gmail.com (A.N.J.)

**Keywords:** adsorption, aggregation, chemical coagulation, CuO nanoparticles, surfactants, wastewater treatment

## Abstract

The widespread use of copper oxide nanoparticles (CuO NPs) and surfactants in various consumer products makes it likely that they coexist in aqueous environments, making it important to study the effects of surfactants on the fate and transport behavior of CuO NPs. The present study aims to investigate the influence of anionic sodium lauryl sulfate (SLS) and nonionic nonylphenol ethoxylate (NPEO, Tergitol NP-9), on CuO NPs adsorption, aggregation, and removal from water by the coagulation process. The result of the sorption study indicates that both surfactants could be adsorbed on the surface of CuO NPs, and that SLS remarkably decreases the ζ potential as well as the hydrodynamic diameter (HDD) of CuO as compared to NP-9. The kinetic aggregation study showed that both SLS and NP-9 reduced the HDD of CuO NPs and retarded the settling rates at surfactant concentrations above 0.015% (w:v) over a 24 h-period. Moreover, enhanced aggregation of CuO NPs was observed in two environmental waters as compared to pure water, which could be related to their high ionic strength. The addition of surfactants in natural waters has been shown to reduce the aggregation and sedimentation of CuO; however, the reductive effect of SLS was more pronounced than that of NP-9. Finally, the coagulation results showed that the removal efficiencies of CuO, Cu^2+^, and the surfactant in all tested waters at optimum ferric chloride dosage reached around 98, 95, and 85%, respectively. Furthermore, the coagulation mechanism revealed that the combination of charge neutralization and adsorptive micellar flocculation (AMF) might be involved in the removal of both pollutants. The results of the present study provide new insight into the environmental behavior of coexisting NPs and surfactants in wastewater treatment processes.

## 1. Introduction

With ongoing rapid development of nanotechnology, engineered nanoparticles (ENPs) have been used in a variety of commercial products and industrial applications. Among the many ENPs available, copper oxide nanoparticles (CuO NPs) are the ones used most frequently in consumer products, industrial applications, antimicrobial agents, and agricultural activities because of their unique structural properties and other physicochemical characteristics [1,2]. Globally, the annual production of CuO NPs was around 570 tons/year in 2014 and is predicted to grow to 1600 tons/year by 2025 [3]. Thus, there are growing environmental concerns that the large-scale production and applications of CuO NPs will inevitably result in the release of these ENPs into the water, which may pose threats to aquatic life and human health [4]. Therefore, it is crucial to study the environmental fate and transport of CuO NPs in the natural water matrices. 

In the natural environment, CuO NPs may dissociate releasing Cu^2+^ ions, and a high concentration of Cu^2+^ is harmful to both humans and aquatic organisms. Previous studies [5,6] reported the toxicity of CuO NPs to aquatic organisms, such as *Fagopyrum esculentum*, *lymphocyte*, *Pseudokirchneriella Subcapitata*, and plants. The significant adverse effect of Cu^2+^ ions on the increased levels of oxidative stress in microalgae and impaired growth of *Triticum aestivum* have also been well reported [7]. Another recent study [8] demonstrated the acute toxic effect of NPs on humans, including damage to DNA structure and cell membranes. The high concentration of released Cu^2+^ ions affects the growth rate of activated sludge, thereby reducing the overall performance of the conventional treatment process [8]. The potential toxicity of ENPs released in the aqueous environment mostly depends on their bioavailability and transportation behavior in water. As a result, it is essential to understand the colloidal stability of CuO NPs in water in order to reduce their associated ecological risks.

The aggregative behavior of ENPs is a crucial factor in assessing their transportation phenomena in natural waters. Environmental parameters, such as the ENPs physicochemical properties (morphology, primary size, and surface charge) and media chemistry (pH, ionic strength, and dissolved organic matter (DOM)) may affect the aggregation and sedimentation process of CuO NPs [9,10,11,12]. The surface potential of CuO NPs is highly dependent on the solution pH, which affects the surface potential through protonation/deprotonation reactions on the NPs surface [9,13]. Previous [14] study have reported that mono (Na^+^, K^+^) and divalent cations (Ca^2+^, Mg^2+^) effectively compress the electrical double layer (EDL), thereby increasing the aggregation rate of ENPs suspension. Higher concentrations of DOM (such as humic acid, fulvic acid, and salicylic acid) could lead to more adsorbed mass of DOM on the NPs inducing electrostatic repulsion, thereby enhance the stability of the NPs [9,10,11]. Moreover, organic pollutants such as surfactants, which are frequently used in household products and industrial processes, can be found in wastewater in higher concentrations [15]. A recent study reported that the concentration of surfactant in the industrial wastewater typically ranges from 1–1000 mg/L [16]. Detergents are harmful to human health and aquatic life, moreover, their presence in water reduces its quality due to their froth formation property. In addition, due to their strong adsorption capacity, surfactants may form stable colloids, even at low concentrations, by lowering the interfacial surface tension among NPs [17]. The presence of surfactant in ENPs suspension might significantly affect the aggregation behavior of CuO NPs, thus altering their mobility and increasing their bioavailability as well as eco-toxicity in natural water matrices. For instance, Godinez et al. showed that sodium dodecyl benzene sulfonate (SDBS) and Triton X-100 impede the aggregation of titanium dioxide (TiO_2_) by enhancing the steric hindrance among the NPs. At alkaline pH (9.0), an increased transport distance of TiO_2_ in a porous medium column has been reported with increasing surfactant concentration [18]. The results of a recent study [19] indicated that lower concentrations of 0.004% (w:v) of detergents such as cetyl trimethyl ammonium bromide (CTAB) and SDBS can effectively disperse and stabilize the graphene ENPs suspension. Moreover, the functional groups present in the surfactant can be sorbed onto the surface of NPs and hinder the sedimentation phenomena by altering their physicochemical properties, thereby enhancing the NPs stability in an aqueous environment [20]. Some researchers have reported a significant effect of surfactant concentration on the fate and mobility of graphene in a saturated porous medium. The enhanced stability of Al_2_O_3_ NPs suspension in the presence of CTAB surfactant has also been demonstrated in a previous study [21]. In addition, SDBS is widely used for the stabilization of ENPs to produce a surfactant-solubilized NPs suspension that is highly mobile in saturated sandy as well as porous media [22]. Therefore, the co-occurrence of CuO NPs and surfactant may increase the release of heavy metal ions (Cu^2+^) in water bodies due to less aggregation and enhanced internalization of CuO NPs, thereby increasing the risks of exposure to aquatic organisms as well as humans, if the source is used as potable water. For these reasons, the removal of CuO NPs and surfactant from aqueous matrices is becoming increasingly important.

Most of the previous studies [9,10,12,20] have only focused on the influence of DOM on the fate, mobility, and removal of ENPs in laboratory-used pure water or synthetic solutions. After an extensive literature search, we found that the interaction between surfactant and CuO NPs in water has barely been touched by the environmental scholars. Moreover, previous studies also seem insufficient regarding the influence of surfactant on the adsorption, aggregation, and simultaneous removal of Cu^2+^ and CuO NPs ions by the chemical coagulation process. Therefore, in this study, we investigated the influences of the two commonly-used surfactants of anionic sodium lauryl sulfate (SLS) and nonionic nonylphenol ethoxylates (NPEO- Tergitol, NP-9), on the adsorption, aggregation, and sedimentation of CuO NPs in various water matrices. We also determined the effects of both surfactants and their concentrations on the simultaneous removal of Cu^2+^ and CuO NPs by the chemical coagulation process from synthetic solutions and natural waters. Furthermore, the obtained data were used to understand the fate, mobility, and interaction mechanism of CuO NPs in water.

## 2. Materials and Methods 

### 2.1. Chemicals

The CuO NPs powder (CAS#1317380, purity ≥97.5%), with vendor reported particle size of 50 nm (TEM), and surface area (BET) of 29.2 m^2^/g (see Supplementary Materials Table S1), as well as two model surfactants, SLS (99+% purity) and NP-9 (97+% purity), were procured from Sigma-Aldrich (St. Louis, MO, USA). Potassium chloride (KCl), magnesium chloride (MgCl_2_), sodium hydroxide (NaOH), hydrochloric acid (HCl), and iron (III) chloride hexahydrate (FeCl_3_·6H_2_O) ACS Reagent Grade were all purchased from local suppliers. Double distilled deionized (DI) water (18.2 MΩ/cm), was produced using the Synergy water system (Milli-Q, Millipore, Burlington, MA, USA) and used to prepare all of the stock solutions.

### 2.2. Stock Solution Preparation

The CuO NPs stock solution was prepared by dispersing 0.1 g of CuO nanopowder into 1 L of DI water, then probe sonicating it for 30 min, as described in our previous study [23]. Then, the CuO stock solution was diluted in order to achieve the desired working solution. The detailed physicochemical properties of both of the surfactants are presented in Supplementary Materials Table S2. The stock solution of 0.1 % (w:v) of each surfactant was prepared in DI water. The measured total organic carbon (TOC) concentrations of SLS and NP-9 stock suspension were 537.05 and 614.57 mg/L, respectively. The 0.1 M stock solution of ferric chloride (FC) coagulant was prepared by dissolving a specific amount of FeCl_3_·6H_2_O into the DI water. The stock solutions were kept in the dark at 4 °C prior to being diluting to the required experimental concentrations.

### 2.3. Experimental Water Samples: Preparation and Characteristics

Four water samples were used to investigate the effect of the surfactant on the environmental behavior of CuO NPs. Two types of water, i.e., freshwater (FW) and domestic wastewater (DWW), were synthetically prepared in the laboratory according to the previously described methods (See Supplementary Materials Section 1.1). Another sample was tap water (TW) collected from the Sungkyunkwan University Suwon Campus (Gyeonggi-do, Korea). In addition, industrial wastewater (IWW) sample was obtained from a metal processing company (LS-Nikko Copper Inc., Onsan National Industrial Complex, Ulsan, Korea). The IWW was diluted so as to reduce the concentrations of heavy metal ions and total organic carbon (TOC). The physicochemical compositions of the synthetic and natural waters are shown in Supplementary Materials Table S3. Moreover, Table 1 shows the detailed parameters after spiking CuO and surfactants in synthetic and natural waters. All of the types of water were filtered through 0.45µm glass fiber filter and stored in the dark at 4 °C prior to experimentation. The pH and TOC of the tested water samples were analyzed using a pH meter (HACH-HQ40d portable multi-parameter meter, Loveland, CO, USA) and TOC analyzer (TOC-5000A, Shimadzu Corporation, Kyoto, Japan). The concentrations of F^−^, SO_4_^2^^−^, Cl^−^, HCO_3_^−^, K^+^, Ca^2+^, Na^+^, and Mg^2+^ ions concentration in the environmental waters were measured through ion chromatography (861-Advanced Compact IC, Metrohm AG, Herisau, Switzerland) using standard methods [24].

### 2.4. Different Batch Experiments 

#### 2.4.1. Batch Adsorption Study and Isotherm Modelling 

In order to better understand the adsorption behaviors of surfactants onto the surface of CuO NPs, adsorption isotherms were obtained from a batch equilibration experiment at pH 7. The stock suspension of CuO NPs was diluted to 50 mg/L, then added to 100 mL vials containing different concentrations (0 to 100 mg/L) of both SLS and NP-9 surfactants. Subsequently, the solution vials were shaken in a shaker (SK, 300 companions, Korea) at 150 rpm for 24 h; following equilibration, the suspensions were centrifuged at 10,000 rpm for 30 min, and then the supernatants were collected for TOC analysis. In addition, in order to elucidate the adsorption mechanism and functional group attachment onto the surface of CuO NPs, Fourier transform infrared analysis (JASCO, FT-IR-4700, Easton, PA, USA) of the CuO-surfactants complexes was conducted. Two commonly-used nonlinear isotherm models, Langmuir and Freundlich, were used to plot the experimental data, as shown in Equations (1) and (2), respectively:

(1) $$q_{e}\;=\;\frac{q_{m}K_{L}C_{e}}{1+K_{L}C_{e}}$$

(2) $$q_{e}=K_{F\;}C_{e}^{\frac{1}{n}}$$

where *q_e_* (mg/g) is the sorbed amount of SLS and NP-9 onto the surface of CuO; *C_e_* (mg/L) is the equilibrium concentration of both surfactants in suspension; *q_m_* and *K_L_* (L/mg) shows the maximum saturated sorption amount and the Langmuir constant relating to binding strength, respectively; and *K_F_* and *n* (L/mg) in the Freundlich equation correspond to the adsorption capacity and intensity of heterogeneity, respectively.

#### 2.4.2. Influence of Surfactants on the Aggregation and Sedimentation of CuO NPs 

The aggregation and sedimentation experiment of CuO NPs suspension was conducted in solutions containing both surfactants. Briefly, the CuO NPs and surfactant stock suspensions were diluted until achieving a 50 mL mixed solution containing 50 mg/L CuO and surfactant (0–0.03%) concentration. Afterward, the ζ potential of CuO NPs in each vial was measured using Zetasizer (Zeta-sizer NanoZS, Malvern, Worcestershire, UK). In order to simulate the dynamic natural water environment, the suspension vials were shaken at 150 rpm for 24 h. Following the completion of the 24 h experiment, an aliquot of 2.5 mL was transferred to a disposable cuvette in order to analyze the average hydrodynamic diameter (HDD) of CuO aggregate in the surfactant solution as measured through the dynamic light scattering (DLS) method. The sedimentation experiments were conducted so as to further elucidate the effects of both surfactants on the environmental transport and fate of CuO NPs. Following the completion of the 24 h experiment in the shaker, an aliquot of CuO NPs suspension containing various concentrations of each surfactant was added into the 3-mL quartz cuvette of an ultraviolet-visible spectrophotometer (UV–Vis Optizen, 2120 UV-Vis, Mecasys, Daejeon, Korea). The CuO NPs concentration was measured at a wavelength of 240 nm, and the UV-Spectra were measured at different time intervals for 24 h. Since absorbance increases with the NPs concentration, the sedimentation rate of CuO can be expressed by C_t_/C_0_, where C_0_ and C_t_ are the absorbances measured at times 0 and t during the sedimentation process, respectively [12]. Following the same procedure, additional aggregation and sedimentation experiments were performed in synthetic and natural waters.

#### 2.4.3. Effect of pH and Ionic Strength on the Aggregation of CuO NPs in the Presence of Surfactants

In natural waters, the pH and ionic strength (IS) are the key parameters affecting the behavior of ENPs in the aqueous environment, so the influences of the surfactants on the aggregation of CuO NPs in solutions with different pH and IS values were also studied. The suspension containing 50 mg/L CuO NPs and specific concentrations (0.015–0.030%) of both surfactants were prepared, and the pH values (5.0 to 9.0) were adjusted using 0.1 M NaOH or HCl solution. A separate set of experiments was carried out to investigate the effects of electrolytes such as monovalent (KCl) and divalent cations (MgCl_2_) on the aggregation kinetics of CuO NPs in the presence of both surfactants. The IS in the case of monovalent (K^+^) ions were set between (0–50 mM), while for divalent (Mg^2+^), the IS were adjusted between (0–5 mM). Following the completion of the 24 h batch experiments, the HDD of the CuO NPs in both cases were measured through DLS.

#### 2.4.4. Chemical Coagulation Experiments

In order to further elucidate the effects of surfactants on the removal behavior of CuO NPs during water treatment, chemical coagulation was conducted in synthetic and natural waters. Prior to the experiments, the CuO NPs suspension was prepared in 1 mM NaHCO_3_ solution, then probe sonicated. The coagulation experiments were conducted in a jar tester (SJ-10, Young Hana Co., Ltd. Gyeongsangbuk-Do, Korea). A predetermined amount of FC coagulant was dosed, and the pH of the suspension was adjusted to 7.0 using 0.1 M NaOH or HCl solutions. The experiments were completed in three steps, as described in our previous study [23]; specifically: (1) rapid mixing at 200 rpm for 2 min to initialize the coagulation, (2) slow mixing at 40 rpm for 20 min to increase flocculation, (3) and sedimentation for 30 min. After settling, a 50 mL aliquot was collected so as to measure the different quality parameters. The effects of various FC dosages (0–0.9 mM) were studied on environmental waters, where 0 represents the control experiment (absence of coagulant) for each tested sample. All of the experiments were performed in triplicate, and the relative standard deviations (RSD) were reported.

### 2.5. Other Analytical Techniques 

An aliquot of 50 mL sample was immediately collected following the coagulation experiment, then centrifuged at 10,000 rpm for 30 min in order to measure the Cu^2+^ and CuO NPs in the solution. The residual CuO NPs concentration was analyzed by measuring the turbidity of the suspension using a turbidimeter (Hach Benchtop 2100N, Loveland, CO, USA). The Inductively Coupled Plasma Optical Emission Spectroscopy (ICP-OES, Agilent Technologies, Santa Clara, CA, USA) was used to measure the metal ions concentration in supernatant. The SLS and NP-9 surfactant were analyzed by liquid chromatography, then a mass spectrometer (MS 4500 Q trap, AB SCIEX, Foster, CA, USA). Raman spectroscopy was conducted with a high-resolution confocal Lab Ram HR Evolution microscope (Horiba Jobin Yvon, Horiba, Bensheim, Germany). Furthermore, the structural characterizations of the CuO powder, such as XRD and XPS, were investigated using Rigaku D max C III, X-ray diffractometry (Rigaku Corporation, Tokyo, Japan), and X-ray photoelectron spectroscopy (XPS) using (XSAM HS, KRATOS, Kratos, UK). 

## 3. Results and Discussion

### 3.1. Characterization of CuO NPs 

**Figure 1:**
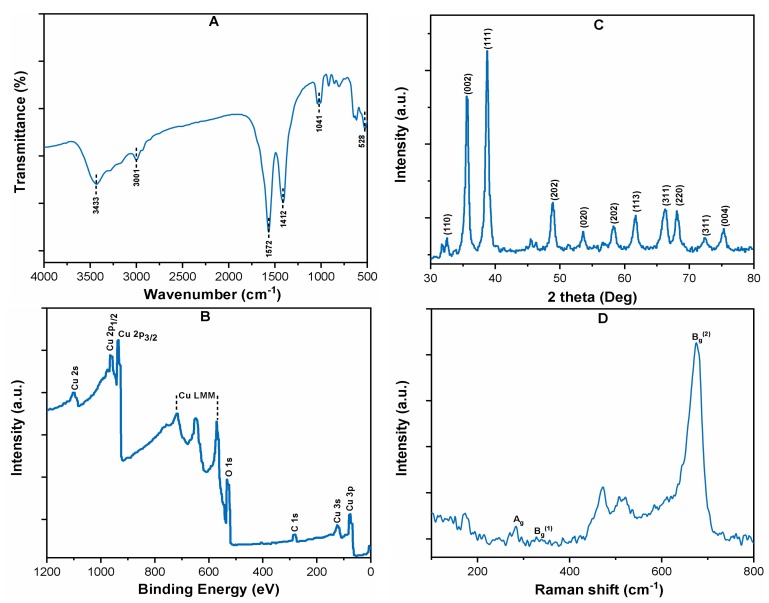
Characterization: (**A**) FT-IR; (**B**) XRD; (**C**) XPS survey spectrum; and (**D**) Raman survey spectrum of CuO powder.

Figure 1A shows the FT-IR spectra of pristine CuO NPs, which indicates the presence of Cu–O stretching vibrations band at 528 cm^−1^ [11]. The crystalline structure of CuO NPs was studied by XRD, and the results are given in (Figure 1B). The diffraction peaks at various angle (32.56, 35.58, 38.74, 48.75, 53.62, 58.32, 63.39, 66.21, 68.18, and 75.19) units correspond to the (110), (002), (111), (202), (020), (202), (113), (311), (220), (311), and (004) planes, indicating the high crystalline structure of CuO (JCPD: 48-1548). The XPS spectrum of CuO is shown in (Figure 1C), which shows the binding energy peaks at 942, 921, and 530 eV, corresponding to Cu 2p_1/2_, Cu 2p_3/2,_ and O 1s, respectively. The lattice dynamics of pristine CuO were analyzed through Raman spectroscopy (Figure 1D). The CuO shows the three Raman active modes of Ag, Bg (1), and Bg (2), which were observed at 283, 515, and 619 cm^−1^. Moreover, a weak peak at about 190 cm^−1^ for the Cu–O structure was also observed in the Raman spectra [24]. The UV-Visible spectrum of CuO was recorded, which showed a strong absorption peak at 240 nm (Supplementary Materials Figure S1A). 

In addition, CuO NPs dispersed in DI water showed an average particle size of (225 ± 38) nm, as measured by DLS, which is much larger than the vendor-reported size (<50 nm) (Supplementary Materials Figure S1B). This may be attributable to the increase in Van der Waals (vdW) forces between the NPs, thus resulting in the formation of large aggregates in solution [24]. The Isoelectric point (IEP) of CuO was determined to be approximately ~8.6 (Supplementary Materials Figure S1C). Our results are consistent with those of previous studies [9,11] which reported that pH_iep_ of CuO was between 6.5–8.8.

### 3.2. Adsorption Study

**Figure 2:**
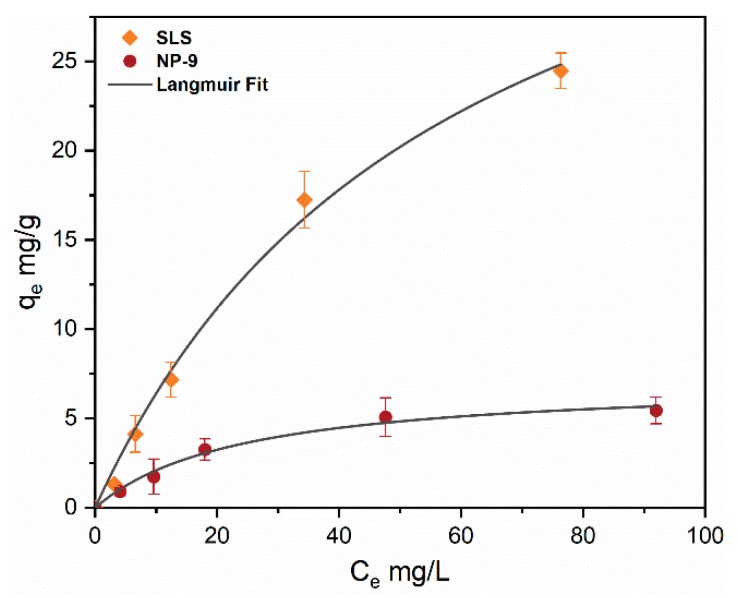
Isotherm modeling (0–100 mg/L) of SLS and NP-9 surfactants onto CuO NPs (50 mg/L) at pH 7.

Figure 2 shows the adsorption isotherms with varying concentrations (0–100 mg/L) of SLS and NP-9 surfactants with CuO NPs concentration (50 mg/L). It can be observed that both of the surfactants adsorbed onto the surface of CuO NPs; however, the SLS surfactant shows a higher adsorption capacity than NP-9. The isotherm of NP-9 reached a plateau at a concentration up to 25 mg/L, showing complete NPs surface coverage. 

Moreover, the adsorption isotherms of both surfactants were better fitted by the Langmuir model (Table 2). This suggests that electrostatic cross-linking may play an important role in the monolayer adsorption of surfactant onto the CuO NPs surface. The fitted q_max_ values of SLS and NP-9 calculated from the equation were 48.84 mg/g with R^2^ values of 0.993 and 7.17 mg/g with an R^2^ of 0.984, respectively. Our results are consistent with earlier studies [20,25] which reported the preferential adsorption of ionic surfactant onto the surface of TiO_2_ and ZnO NPs. Moreover, SLS contained a higher content of sulfate species and was considered to be more hydrophilic as compared to NP-9 surfactant. These results also indicate that the adsorption of SLS on the surface of CuO NPs may occur via H-bond interaction between the oxide surface and sulfate head functional groups [19]. The electrostatic cross-linking forces might be responsible for the adsorption of NP-9 onto CuO attributable to the relatively high binding affinity of Cu^2+^ with a phenol group. The strong hydrophilic and electrostatic attractive forces present in SLS surfactant may provide favorable adsorption sites for their molecules on the surface of CuO NPs. This strong adsorption of SLS could be attributed to electrostatic interactions between the negatively-charged sulfonate head and the positively-charged CuO NPs [20]. These results are consistent with the observation of a previous study [26] that anionic surfactants have a higher affinity to NPs than nonionic surfactants due to their bilayer adsorption property on the colloid surface. In general, such observations suggested the monolayer adsorption of surfactant was formed on CuO NPs.

### 3.3. Effects of the Surfactants on the ζ Potential and HDD of CuO NPs

**Figure 3:**
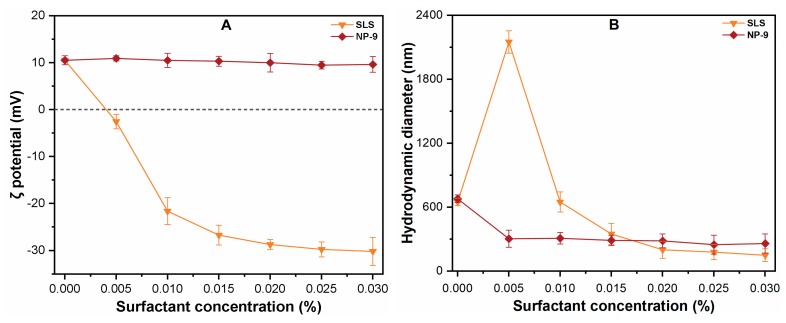
Effects of surfactants concentration (0–0.030%) on the (**A**) ζ potential; (**B**) hydrodynamic diameter of CuO NPs suspension at pH (7.0 ± 0.1).

Figure 3 shows the effect of surfactant on the ζ potential and HDD of CuO NPs in aqueous solutions. The figure shows that the ζ potential of CuO NPs decreased from +12.5 to –31.4 mV with an increase in SLS concentration from 0 to 0.030%. However, NP-9 showed an insignificant effect on the surface potential of CuO NPs, even at higher concentrations, which was consistent with the results of an earlier study [26]. The addition of surfactants also significantly affected the HDD of CuO NPs and most of NPs aggregated in solutions after shaking for 24 h. In the absence of both surfactants, the increase in HDD (650 ± 45 nm) of CuO NPs was observed after a continuous shaking process, which indicated that shaking enhanced the collision frequency and facilitated the aggregation of CuO NPs. By contrast, when the SLS concentration was 0.005%, the ζ potential of CuO approached zero, and HDD was found to be 2100 ± 224 nm. The increased size of the particle may be related to the reduced absolute potential of CuO NPs (Figure 3A). These results are consistent with previous studies [20,26] which reported that the appropriate concentration of SLS decrease the absolute potential of particle, which then reduces the electrostatic repulsive forces among NPs, thereby increase the aggregates size. With further increasing SLS concentrations (0.05–0.030%), an increase in ζ potential value in a consistently negative trajectory (–2.5 to –32.85) mV was observed. The HDD of CuO at an SLS concentration of 0.030% was recorded as 235.8 ± 65 nm, which was much smaller than that measured in DI water. This may be attributable to the fact that when the absolute surface charge of the particle exceeds ± 20 mV, the NPs tend to repel each other, thereby remaining stable in the solution for a longer period [18]. 

By contrast, the addition of NP-9 in suspension reduced the HDD of CuO NPs without having any noticeable effect on the absolute surface charge throughout the tested (0–0.030%) concentration. The adsorption of non-ionic surfactants such as NP-9 onto CuO NPs mainly occurs via non-dissociable phenol, ester, and alkylbenzene groups as well as phenol ring structures [19]. Thus, the attachment of NP-9 on the CuO NPs surface enhances the effect of steric inhibition, thereby reducing the size of aggregates during the batch experiment. As the NP-9 concentration reached 0.010%, the aggregate size of CuO was reduced to 298 ± 48 nm, which was 50% less than the size observed in DI water. These findings confirm the high capacities of both surfactants to enhance the colloidal stability of CuO NPs suspension.

### 3.4. Effects of Surfactants on the Aggregation Kinetics of CuO NPs

**Figure 4:**
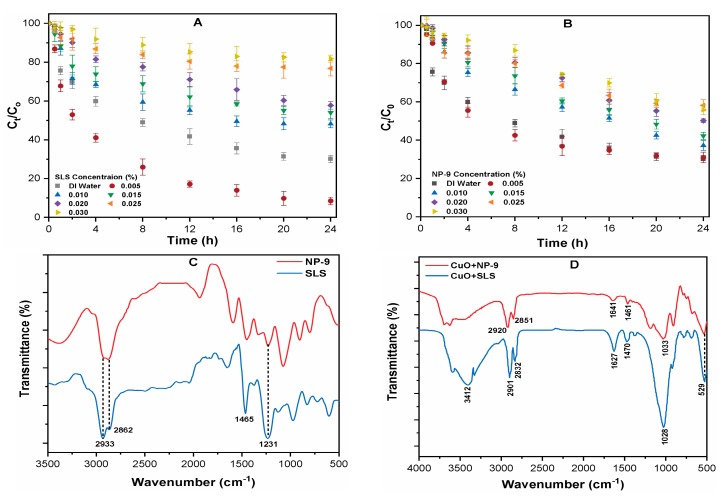
Effects of surfactants concentration (0–0.030%) on the aggregation rate of CuO NPs in the presence of (**A**) SLS; (**B**) NP-9; and FT-IR spectra showing (**C**) pristine surfactants and; (**D**) CuO-surfactants complex.

In order to further elucidate the influence of surfactant on the sedimentation of CuO NPs, the rate of aggregation was measured with a UV-Vis spectrophotometer at a specific wavelength of 240 nm. Prior to the measurement of CuO NPs suspension, the absorbances of both surfactants were recorded at a similar wavelength in order to reduce any interfering effect. It can be observed that CuO NPs aggregates rapidly in the DI water and that only 30.4% remained suspended following the completion of the experiment (Figure 4A,B). The aggregation of CuO was substantially enhanced in the presence of SLS at 0.005% concentration, leaving only 4.49% of the NPs in the above solution after 24 h. Higher SLS concentrations (0.015%) significantly retarded the rate of CuO NPs sedimentation, and more than 85% NPs remained stabilized in the suspension after 24 h (Figure 4A). The effect of SLS on the aggregation of CuO is consistent with our particle size result. At SLS concentrations above 0.015%, the adsorption of anionic surfactant molecules impedes the aggregation of CuO NPs and slows the settling rate of CuO NPs. A concentration-dependent effect of NP-9 surfactant on CuO NPs sedimentation was observed during the experimental period (Figure 4B). The higher concentration 0.030% NP-9 retarded the aggregation of CuO, and as high as 48% stable NPs remained in the suspension after 24 h. In summary, the anionic surfactant (SLS) have a more pronounced adverse effect on the aggregation of CuO NPs than nonionic surfactant (NP-9). 

In addition, the FT-IR spectra of pristine surfactant and CuO-surfactant complexes were analyzed in order to explore the functional group attachment on the surface of CuO NPs (Figure 4C,D). The peaks around the 3400–2800 cm^−1^ regions are ascribed to the asymmetric and symmetric stretching of CH_3_, CH_2_, and OH bonds [27]. The bands at 1465 and 1231 cm^−1^ correspond to the bending of CH_2_ and C-O stretching along with OH deformation in COOH, respectively [28]. The asymmetric doublet peak for OSO_3_ stretching in pristine SLS shifted down in frequency at 1028 cm^−1^, and it appeared in the spectrum of SLS adsorbed CuO complexes [29]. This significant shift in wavenumber indicates that the primary adsorption mechanism may involve hydrophobic and electrostatic interactions [30]. The appearance of certain bands and shift in peaks of the CuO-surfactant complexes confirms that the surfactant made enough of an impact on the metal oxide surface. Moreover, the disappearance and significant shift to 1033 cm^−1^ in the (CuO+NP-9) spectra further suggests the attachments of the hydrophilic tail and phenol head [20,30]. Therefore, the presence of surfactant may increase the environmental risk of CuO NPs by altering their mobility and fate in the water matrices. 

### 3.5. Effect of Surfactants on HDD of CuO with Various pH and Electrolyte Types

**Figure 5:**
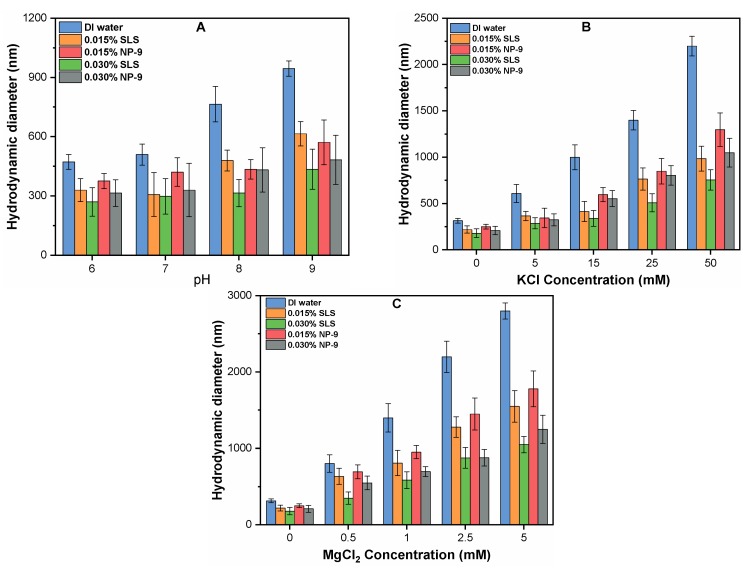
Effect of surfactants concentration (0–0.030%) on HDD of CuO NPs as a function of (**A**) pH; (**B**) KCl; and (**C**) MgCl_2_.

The solution chemistry may affect the properties of the CuO NPs, such as their solubility, particle size, and surface potential. Thus, experiments were conducted at varying pH (6–9) of media containing ranges of (0.015–0.030%) of SLS and NP-9 (Figure 5A). The results indicated that, as the initial pH of the solution increased from 6 to 9, the average particle size of CuO aggregate was enhanced from 550 ± 68.2 to 1040.1 ± 205.4 nm. A significant increase in the HDD of CuO was observed between 8 to 9, which may be attributed to the closeness of the zero point of charge of the solution. The NPs have zero/negligible surface potential, thus significantly weakening the electrostatic repulsive forces between the particles, thereby increasing the size of NPs [12,14]. However, the addition of surfactant significantly retarded the growth of CuO aggregates. For instance, in the absence of both surfactants, the aggregate size of CuO at pH 9 was found to be 1040.1 ± 205.4 nm after 24 h. However, the HDD of CuO NPs in the suspension containing 0.030% SLS and NP-9 were reduced to 51.31 and 38.24%, respectively (Figure 5A). This may be attributable to the electrostatic steric hindrance effect of surfactant molecules, which reduces the size of the CuO NPs in suspension [26]. Moreover, the effect of SLS on the size reduction of CuO was more pronounced than that of NP-9 surfactant.

Figure 5B,C present the influence of surfactant on the aggregate size of CuO NPs as a function of various concentrations of mono and divalent cations. The aggregate size of CuO NPs was significantly enhanced at higher concentrations of both electrolytes. For instance, at the higher tested concentration of KCl (50 mM) and MgCl_2_ (5 mM), the aggregate sizes of CuO NPs were observed to be 2285.7 ± 345.1 nm and 2854.3 ± 345.1 nm, respectively, after 24-h batch experiments. This aggregation behavior is consistent with earlier studies [12,27], which reported that higher concentrations of cations reduce the electrostatic repulsion forces and sink the energy barrier among the NPs. Moreover, the higher ionic strength of monovalent cation compresses the EDL of NPs, thereby promoting the agglomeration of NPs in suspension. Note that a lower concentration of divalent cations substantially increases the aggregate size of CuO due to the simultaneous effects of charge neutralization and EDL compression [14]. However, the addition of surfactant significantly reduced the aggregate size of CuO in suspension. The presence of 0.030% of SLS and NP-9 surfactants in the suspension containing 15 mM KCl reduces the sizes of the CuO NPs to 65 and 45%, respectively. The effect of the 0.030% concentration of both SLS and NP-9 in water with 1 mM MgCl_2_ reduced the size of CuO NP by 54 and 50%, respectively. With a further increase in KCl and MgCl_2_ concentration to 50 and 5 mM, the sizes of CuO NPs were decreased to 69.4 and 56.8 %, and 66.1 and 57.6%, respectively. 

### 3.6. Effects of Surfactants on the Aggregation Behavior of CuO NPs in Environmental Waters

**Figure 6:**
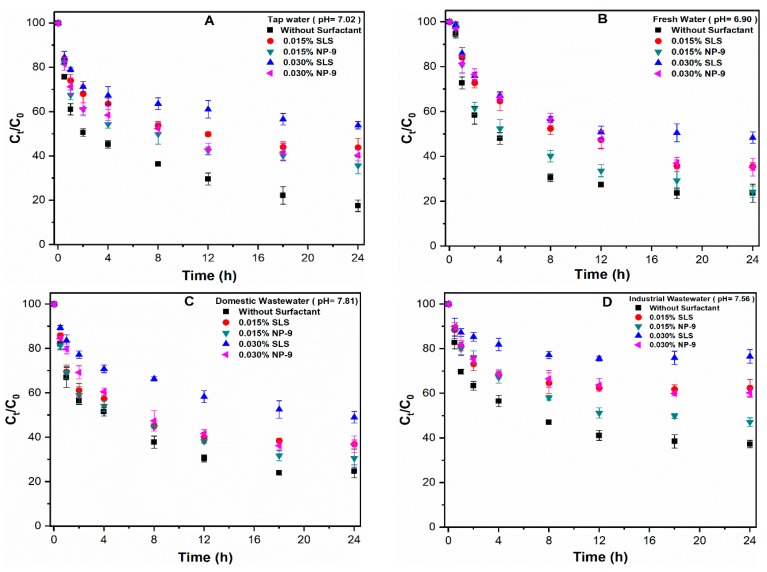
Effects of SLS/NP-9 concentration (0.015 and 0.030%) on the aggregation of CuO NPs in (**A**) tap water; (**B**) fresh water; (**C**) domestic wastewater; and (**D**) industrial wastewater.

Figure 6 presents the influence of the surfactant type (SLS and NP-9) and concentration (0.015 and 0.030%) on the aggregation behavior of CuO NPs in natural water matrices. The results indicate that the addition of both surfactants hinders the sedimentation of CuO NPs in all studied types of water. Moreover, the discrepant aggregation behavior of CuO NPs in the presence of SLS was observed in all of the natural waters. These results are consistent with those of an isotherm study which showed the strong adsorption potential of SLS molecules on the surface of CuO NPs (Figure 2), resulting in reduced aggregation. Upon the additions of (0.030%) SLS and NP-9 in tap water, around 58.76 and 40.23% of CuO NPs remain stabilized in the solution after 24 h, respectively (Figure 6A). By contrast, the measured values at the same surfactant concentration in DI water were 80.01 and 58.15%, respectively. Our observation is consistent with a previous study [9] which reported that many parameters, i.e., pH, DOM, and IS, may affect the aggregation behavior of CuO NPs in the aqueous solution. 

Moreover, noticeable changes in the aggregation behavior of CuO NPs were observed in fresh water and domestic wastewater (Figure 6B,C). The stable CuO NPs aggregates in the presence of 0.030% SLS and NP-9 in fresh and domestic wastewater were recorded to be 50.24 and 38.9%, and 52.12 and 36.61%, respectively. The similar aggregation trend observed in both waters may be attributable to their water chemistry. As shown in Supplementary Materials Table S2, fresh water presented a much lower DOM concentration than surfactants in NPs suspension; for this reason, the competitive sorption behavior between DOM and surfactants becomes negligible. By contrast, the steric hindrance effects of DOM and surfactants molecules might be reduced due to the presence of high electrolyte concentration in domestic wastewater [10,17]. Moreover, the cations present in these media may interact with DOM and form intermolecular bridging between organic pollutants and CuO NPs, resulting in hetero-aggregation [11,20]. Our results are consistent with those of the previous study [26] that reported that higher IS may effectively compress the EDL of particles, thus reducing the electrostatic repulsive forces among NPs, thereby enhancing the sedimentation rate. In addition, the slightly higher sedimentation in domestic wastewater than fresh water may be attributable to the increased interaction between negatively-charged SLS molecules and positively-charged cations. The significant reductions in the aggregation of CuO NPs in the presence of (0.030%) SLS and NP-9 in industrial wastewater were observed to be 80.21 and 65.73%, respectively (Figure 6D). The higher TOC content in these media facilitate the competitive sorption phenomena between DOM and surfactant molecules. In addition, the interfering ions such as arsenic (As) and antimony (Sb) may also contribute to the stabilization process of NPs in these waters. However, in order to confirm this effect, a separate set of experiments are required to explore the effect of heavy metal ions on the aggregation of NPs in real wastewater. Moreover, the aggregate size of CuO NPs was remarkably reduced in the presence of SLS surfactants in all environmental waters (Figure S2). In general, higher concentrations of both surfactants affect the aggregation phenomena of CuO NPs; however, the effect of ionic surfactants on aggregation was much higher than that of non-ionic surfactants.

### 3.7. Removal of Contaminants from Environmental Waters

**Figure 7:**
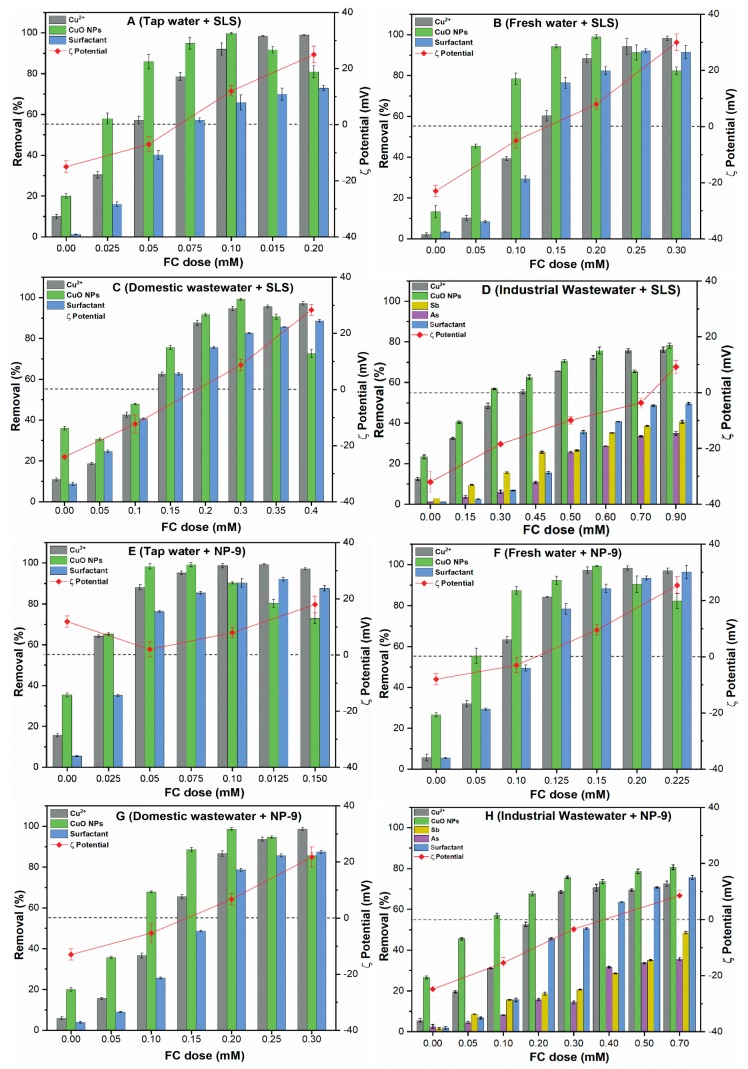
Removal of CuO NPs, Cu^2+^, surfactant, from environmental waters (**A–H**), as a function of FC coagulant dosage with corresponding ζ potential.

Figure 7 presents the removal of CuO, Cu^2+^, and surfactants from environmental waters by chemical coagulation with different FC dosages. The results indicate that the contaminant removal depends highly upon the type and characteristics of the studied waters, as shown in Figure 7A–H. It can be observed that, without coagulant, only 20–40% CuO NPs removal was achieved in all of the studied waters, while the removal was found to be higher in tap water and domestic wastewater. This may be related to the presence of divalent cations in these waters, which compress the EDL of the CuO particles and reducing the EDL electrostatic force between them, thereby enhancing the flocculation [31]. In addition, the reduced removal efficiencies of Cu^2+^ and surfactants were observed in the absence of coagulant in all of the tested waters. 

The addition of FC coagulant increases the removal efficiencies of the contaminants in all of the environmental waters (Figure 7A–H). The FC results in the formation of ampler composite flocs until reaching a plateau, i.e., ~98% removal. The tap water containing both surfactants required a lower FC dosage between 0.05-0.10 mM in order to achieve higher NPs removal. Moreover, a slightly higher FC (0.01–0.20 mM) dose was required in the case of fresh water to achieve a similar removal efficiency. This may be related to the particle size, since the CuO NPs dispersed in tap water and freshwater containing both surfactants showed a larger size as compared to the remaining studied waters (Table 1). By contrast, the domestic and industrial wastewaters require a larger FC dosage between (0.2–0.9 mM) to achieve the maximum removal efficiency 85–90 % of NPs. This suggests that the size of NPs played a significant role in the determination of optimum dosage (OD); for example, the larger NPs have a smaller diffusion coefficient and thereby require a lower FC dosage than smaller NPs. Moreover, the electrostatic interactions between the CuO NPs and coagulant may be reduced due to the presence of high molecular weight (HMW) compounds in domestic and industrial wastewaters. In all of the environmental waters, following OD, an additional coagulant does not improve the removal efficiency, since it increases the solution turbidity and enhances the ζ potential of CuO NPs. These results are consistent with those of previous studies [23,32] that reported that the charge inversion and restabilization of coagulated NPs occur due to the presence of excess polyelectrolytes in the solution. 

In all environmental waters, the relatively less removal of Cu^2+^ ions was observed at lower FC dosage, due to less availability of FC active sites in the solution. Interestingly, higher removal up to 90–95% of Cu^2+^ were observed at OD in all tested waters (Figure 7A–D). The heavy metals in cationic form may be linked with the precipitate matrix colloids through the interaction mechanism recognized as occlusion [33]. These results are consistent with earlier literature [34] and clarify the influence of electrolyte on Cu^2+^ removal from the complex waters. Moreover, these results further suggest that at lower coagulant dose, the interparticle bridging did not form due to the inadequate compression the EDL of NPs. In contrast, the industrial wastewater presented discrepant removal behavior, where around 28% of Cu^2+^ and CuO NPs remains in the supernatants even at higher FC dosage (0.7–0.9 mM) (Figure 7D,H). The industrial wastewater contains highly reactive metal ions, thus inducing the high surface potential of CuO NPs in these media (Table 1). The concentration of metal ions (Sb, As) in industrial wastewater after coagulation was further analyzed to assess the competitive inhibition effect of these ions on the overall removal process. The results indicated the adverse effect of As and Sb on the simultaneous removal of CuO NPs and Cu^2+^. The presence of other interfering ions induces less charge neutralization, sorption, and interparticle bridging between NPs and FC surface. The most of CuO NPs spiked in the environmental waters containing surfactant have a negative surface charge (−15.4 ± 0.5 and −32.3 ± 0.8) and smaller particle size except tap water with NP-9. Moreover, the addition of higher FC dosage (up to 0.9 mM) enhance the removal of Cu^2+^ (60–80%), Sb (35–43%), and As (38–40%) in industrial wastewater containing both surfactants. A recent study [35] reported that removal efficiencies remarkably decrease in heterogenous complex waters, i.e., industrial and domestic wastewaters due to their variable origin. Nevertheless, the results show that the competitive inhibition effect of Sb, As, species is because of their strong adsorption potential on active FC surface sites.

In the natural environment, the Fe hydrolyzed products effectively bind to CuO surface, thereby reducing the ζ potential of the solution. A similar observation has been reported in the earlier study [36], where NPs practically neutralized and effectively removed when ζ potential value ranges between ±10 mV. Our results suggested that at OD, most of the colloids destabilize and bridging flocculation occurs due to an increase in electrostatic attractive forces among the NPs. Moreover, surfactant might adsorb onto the NPs surface via electrostatic interaction and increase the CuO NPs transportation, as already observed in Section 3.6. At OD, the stronger enmeshment effect of coagulant reduces the steric forces between the NPs, thereby play an essential role in NPs removal from suspension. In summary, the mechanism such as a combined effect of charge neutralization and sorption on FC surface might be involved in simultaneous removal of these contaminants during the chemical coagulation process. However, the removal mechanism might vary according to the variable origin of media and presence of other competitive ions.

### 3.8. Removal of SLS and NP-9 from Environmental Waters

The presence of various surfactants in natural waters might affect the overall removal performance of chemical coagulation process. The removal of surfactants content reached up to 80–90% in all studied waters, except industrial wastewater, where around 40% removal was achieved even at higher coagulant dose (Figure 7). The anionic organic pollutants such as SLS with (pKa = 5.7) could efficiently be removed with Fe^3+^ ions from natural waters. However, dissolution of Fe precipitates might occur in industrial wastewater due to the solubilization capacity of DOM and surfactants complex. Moreover, the addition of surfactants in such water increase the stability of NPs by altering the ionization state and surface properties. Stackelberg et al. also reported the lower removal (40%) of surfactants from water containing other organic pollutants [35]. The hydroxyl groups of FC may react with polarized groups of surfactants through hydrophobic interaction, thereby producing more hydrophilic surface [37]. At OD, the measured ζ potential was found around ±10 mV, which further explain the role of surface potential in the removal phenomena. However, the removal decreases with further increase in the magnitude of ζ-potential (>±30 mV). A previous study [38] reported that most of NPs could be effectively removed between ζ potential window (+3 and −10 mV). In addition, the Stern-diffuse layer may be compressed due to the high concentration of Fe^3+^ ions, thus allowing more sorption of organic micropollutants complexes. The increase in FC dosage reduces the ζ potential of the remaining solution, resulting in the effective removal of contaminants via adsorptive micellar flocculation (AMF) [20,37]. Therefore, in combination with charge neutralization, the AMF mechanism might also be involved in the removal of surfactants and DOM from these waters. These results revealed that characteristics of surfactants, their concentration, and other interfering ions appear to be influential factors in affecting the removal behavior of contaminants in natural waters.

### 3.9. Study Significance

This study is the first-time approach to provide some insight into the adsorption, aggregation, and removal behavior of CuO NPs in waters containing different surfactants. Earlier studies by [16,18,20,25,26] have described the influence of surfactant on the specific parameters of ENPs in the water environment. Moreover, yet no study has been conducted that describes the effect of various surfactants on the removal behavior of CuO NPs from real industrial wastewater. The present research was conducted on two synthetics and two real waters with different characteristics and surfactant concentration. The results showed that the adsorption of anionic surfactant onto CuO improve the colloidal stability through steric hindrance. Thus, lesser aggregation of CuO NPs may increase the risk of release of heavy metal ions, thereby may also alter the NPs fate, bioavailability, and the potential effect on biota. Moreover, the higher concentration of metal ions presents in wastewater, such as As and Sb reduced the coagulation efficiency due to the competition for available active FC sites. This study underscores the importance of understanding the influence of ionic and non-ionic surfactants on the fate and mobility of CuO NPs in the real water and wastewater environment.

## 4. Conclusions

In this study, we investigate the influence of anionic sodium lauryl sulfate (SLS) and nonionic nonylphenol ethoxylate (NPEO, Tergitol NP-9), on CuO NPs adsorption, aggregation, and removal from natural waters by chemical coagulation process. The results revealed that surfactants could be adsorbed on the surface of CuO with the stronger sorption capacity of SLS than NP-9. Moreover, SLS significantly reduced the ζ potential and aggregate size thus hindering the NPs sedimentation. The kinetic aggregation results indicated that the higher 0.030% concentration of both surfactants retarded the aggregation and sedimentation of CuO NPs in synthetic waters. The presence of both surfactants in environmental waters resulted in the adverse effect on the aggregate size of CuO NPs, while the effect of SLS was more pronounced than NP-9. The results also indicated that, at optimum FC dosage, the removal efficiencies of CuO NPs, Cu^2+^, and surfactants were more than 98, 95, and 85%, respectively, in all studied waters. However, the presence of higher concentration of metals ions in industrial wastewater significantly inhibited the coagulation efficiencies of Cu^2+^, CuO, and surfactant up to 20, 25, and 50%, respectively. The mechanisms, such as charge neutralization and adsorptive micellar flocculation (AMF), may be involved in the removal of NPs composite pollutant by chemical coagulation. These findings showed that surfactants enhance the colloidal stability of CuO NPs in natural waters, thereby influence the fate, mobility and removal performance of NPs during the water treatment process. 

## Figures and Tables

**Table 1:** Characteristics of synthetic and environmental waters.

Surfactant	Conc.: (w:v) %	Water Code	pH	CuO NP (mg/L)	Released Cu^2+^ (mg/L)	HDD (nm)	ζ Potential (mV)
Control	0	Control	7.02 ± 0.01	8.74 ± 0.41	0.989 ± 0.12	225 ± 38	12.5 ± 2.5
SLS	0.030	Tap water	6.91 ± 0.03	8.65 ± 0.11	0.995 ± 0.23	257 ± 60	−15.5±1.1
		Freshwater	6.83 ± 0.14	7.87 ± 0.43	1.103 ± 0.13	205 ± 84	−19.1 ± 0.9
		Domestic Wastewater	7.16 ± 0.25	6.19 ± 0.13	2.029 ± 0.07	235 ± 68	−23.8 ± 0.5
		Industrial Wastewater	7.67 ± 0.31	5.17 ± 0.06	3.184 ± 0.01	185 ± 48	−32.3 ± 0.2
NP-9	0.030	Tap water	6.95 ± 0.21	8.05 ± 0.14	0.784 ± 0.28	265 ± 55	11.9 ± 0.5
		Freshwater	6.82 ± 0.01	8.26 ± 0.08	0.996 ± 0.30	314 ± 35	−5.6 ± 0.1
		Domestic Wastewater	7.36 ± 0.14	6.09 ± 0.08	2.719 ± 0.02	245 ± 67	−13.8 ± 0.3
		Industrial Wastewater	6.75 ± 0.11	7.16 ± 0.01	2.769 ± 0.03	205 ± 65	−24.8 ± 0.2

NP: nanoparticles. HDD: hydrodynamic diameter. Conc. Concentrations.

**Table 2:** Langmuir and Freundlich fitting parameters for SLS and NP-9 adsorption onto CuO NPs.

Langmuir Fitting				Freundlich Fitting		
Surfactant	*K*_L_ (L/mg)	*q*_max_ (mg/g)	*R* ^2^	*K*_F_ (mg/g)(L/mg)^1/n^	*n*	R^2^
**SLS**	0.017 ± 0.003	48.84 ± 4.42	0.993	1.367 ± 0.42	1.482 ± 0.17	0.973
**NP-9**	0.041 ± 0.010	7.17 ± 0.53	0.984	0.747 ± 0.25	2.185 ± 0.40	0.934

## Supplementary Materials

The following are available online at https://www.mdpi.com/1660-4601/16/7/1260/s1, Figure S1: (A) UV-Vis spectra of (10 mg/L) CuO NPs in DI waters; (B) Size distribution by the intensity of CuO NPs in DI; (C) ζ potential of CuO NPs as a function of pH; Figure S2: (A) Size ratio of CuO NPs with and without surfactant in various environmental waters; Table S1: Physicochemical properties of CuO NPs used in the current study; Table S2: Properties of surfactants used in the present study; Table S3: Natural and synthetic water characteristic.
