# Supplementary material for: The Influence of Ionic and Nonionic Surfactants on the Colloidal Stability and Removal of CuO Nanoparticles from Water by Chemical Coagulation
Source: Int J Environ Res Public Health. 2019 Apr 9;16(7):1260. doi: 10.3390/ijerph16071260 (PMC6479800; doi:10.3390/ijerph16071260)
Supplement: Supplementary file 1 [file ijerph-16-01260-s001.pdf]

Supplementary Information

The Influence of Ionic and Nonionic Surfactants on the Colloidal Stability and Removal of CuO Nanoparticles from Water by Chemical Coagulation

Rizwan Khan [1], Muhammad Ali Inam [1], Sarfaraz Khan [2], Andrea Navarro Jiménez [2], Du Ri Park [1] and Ick Tae Yeom [1,*]

[1] Graduate School of Water Resources, Sungkyunkwan University (SKKU) 2066, Suwon 16419, Korea; rizwankhan@skku.edu (R.K.); aliinam@skku.edu (M.A.I.); enfl8709@skku.edu (D.R.P.)

[2] Key Laboratory of the Three Gorges Reservoir Region Eco-Environment, State Ministry of Education, Chongqing University, Chongqing 400045, China; Sfk.jadoon@yahoo.com (S.K.); andrenavarrojimenez@gmail.com (A.N.J.)

* Correspondence: yeom@skku.edu; Tel.: +82-31-299-6699

Received: 15 March 2019; Accepted: 7 April 2019; Published: date

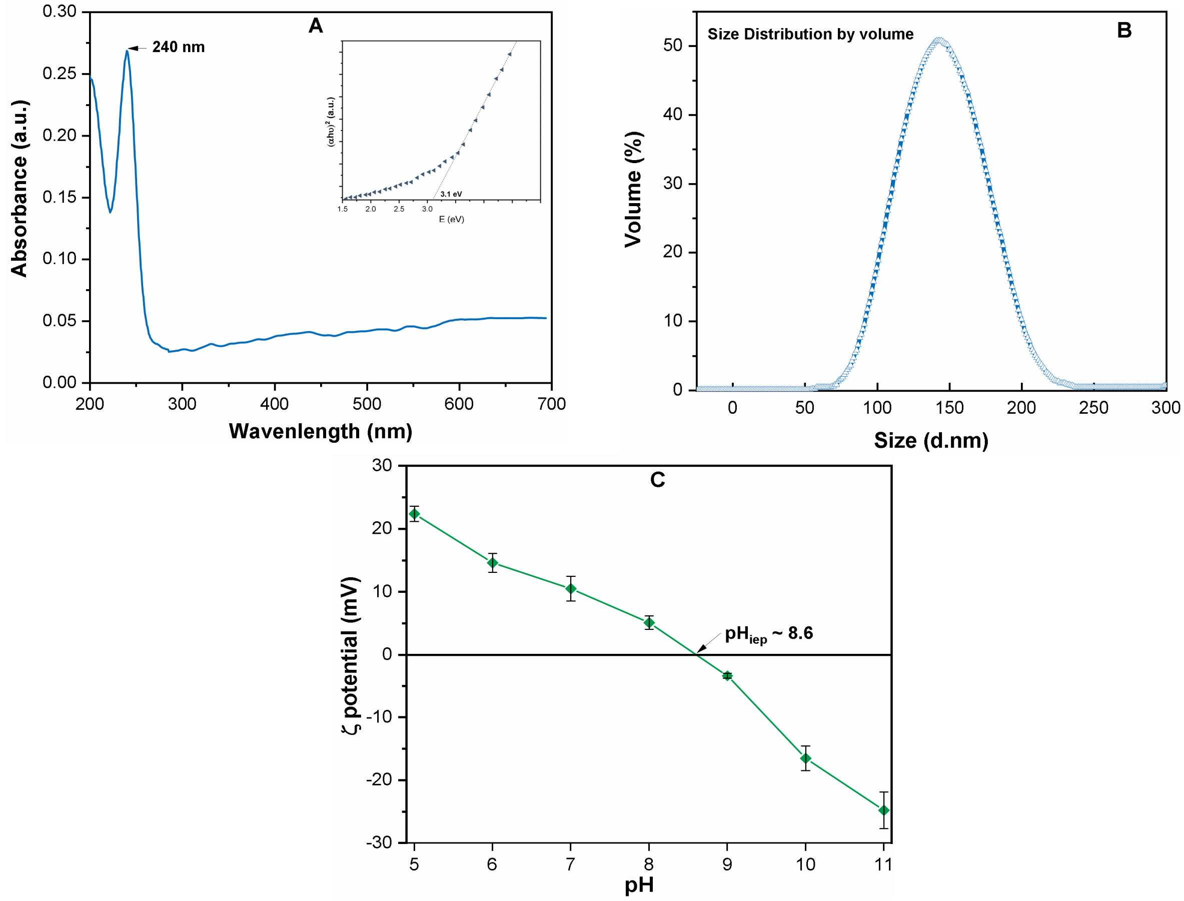

Figure S1. (A) UV-Vis spectra of (10 mg/L) CuO NPs in DI waters; (B) Size distribution by the intensity of CuO NPs in DI; (C) ζ potential of CuO NPs as a function of pH.

Table S1. Physicochemical properties of CuO NPs used in the current study.

Parameter	Unit	Value
Density	g/cm^3	6.372
Vendor-reported size	nm	<50
DLS HDD measured in DI water (n = 10) see Figure S1B	Nm	225±38
BET specific surface area measured (n = 3)	m^2/g	29 ± 3
pH$_{iep}$ see Figure S1C		8.6
Zeta potential in DI water (pH = 7)	(mV)	+12.5 ± 1.6
Purity by ICP-MS	wt %	98.81
Moisture content by TGA	wt %	1.15

Table S2. Properties of surfactants used in the present study.

Surfactant Type	Molecular weight (g/mol)	Chemical Structure	Formula
SLS (Anionic)	288.38	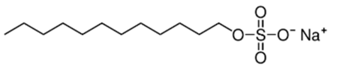	$CH_3(CH_2)_{11}OSO_3Na$
NP-9 (Nonionic)	616.82	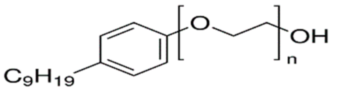	$C_9H_{19}C_6H_4(OCH_2CH_2)_9OH$

Materials and methods

1.1. Synthetic water preparation

The synthetic freshwater and domestic wastewater were prepared in the DI water according to previously described methods [1–4]. All salts used were ACS reagent grade and purchased from local suppliers. Before use all waters were filtered through 0.45 μm glass fiber filter and stored in the dark at 4 °C.

Table S3. Natural and synthetic water characteristic.

Parameter	Unit	Tap water	Fresh water	Industrial Wastewater	Domestic Wastewater
pH [a]	-	7.02	6.90	7.56	7.81
Conductivity [a]	us/cm	82.42	119	619	2280
IS	mM/L	0.002	0.79	8.90	34.0
TOC	mg/L	ND	4.5	35	25
HCO_3	mg $CaCO_3$ /L	>80	12	-	56
PO_4	mg/L	-	0.64	ND	2.71
Na^+	mg/L	0.31	0	15.0	325.3
K^+	mg/L	0.06	1.20	7.53	38.59
Cu	mg/L	-	0	0.39	0.08
Fe	mg/L	-	0	ND	0.35
Mg^{2+}	mg/L	0.19	3.49	27.1	77.0
As	mg/L	-	0	68.52	0
Ca^{2+}	mg/L	0.81	1.50	16.11	119.90
Cl^-	mg/L	0.24	6.61	22.40	501
SO_4^{2-}	mg/L	-	0	10.52	310
Sb	mg/L	-	0	58.77	0

-: Not Measured, ND = Not detected, [a] Measured in lab.

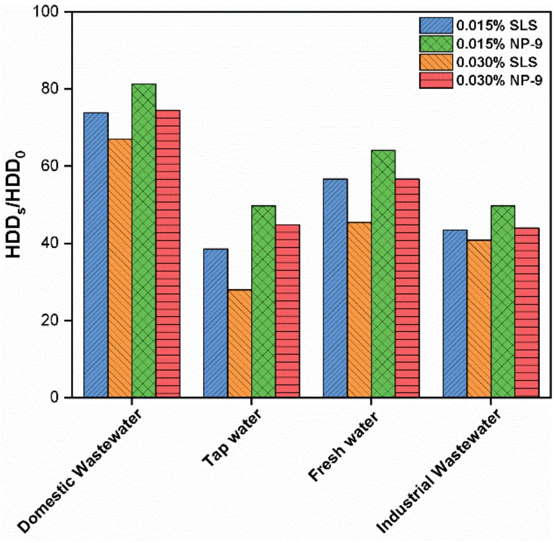

Figure S2. Size ratio of CuO NPs with and without surfactant in various environmental waters.

References

1. Dickson, A.G. Thermodynamics of the dissociation of boric acid in synthetic seawater from 273.15 to 318.15 K. *Deep Sea Res. Part A. Oceanogr. Res. Pap.* **1990**, *37*, 755–766.
2. Marine Biological Laboratory (Woods Hole, M..; Cavanaugh, G.M. *Formulae and methods IV [i.e., 4th ed.] of the Marine Biological Laboratory Chemical Room.* ; Woods Hole, Mass., 1956;
3. Schnabel, W.E.; Dietz, A.C.; Burken, J.G.; Schnoor, J.L.; Alvarez, P.J. Uptake and transformation of trichloroethylene by edible garden plants. *Water Res.* **1997**, *31*, 816–824.
4. United States Environmental Protection Agency Short-term Methods for Estimating the Chronic Toxicity of Effluents and Receiving Waters to Freshwater Organisms Fourth Edition October 2002. **2002**, 1–350.

© 2019 by the authors. Submitted for possible open access publication under the terms and conditions of the Creative Commons Attribution (CC BY) license (http://creativecommons.org/licenses/by/4.0/).
